# Evaluation of Beneficial Metabolic Effects of Berries in High-Fat Fed C57BL/6J Mice

**DOI:** 10.1155/2014/403041

**Published:** 2014-01-14

**Authors:** Lovisa Heyman, Ulrika Axling, Narda Blanco, Olov Sterner, Cecilia Holm, Karin Berger

**Affiliations:** ^1^Department of Experimental Medical Science, Lund University, Lund, Sweden; ^2^Centre for Analysis and Synthesis Organic Chemistry, Department of Chemistry, Lund University, P.O. Box 124, 22100 Lund, Sweden

## Abstract

*Objective*. The aim of the study was to screen eight species of berries for their ability to prevent obesity and metabolic abnormalities associated with type 2 diabetes. *Methods*. C57BL/6J mice were assigned the following diets for 13 weeks: low-fat diet, high-fat diet or high-fat diet supplemented (20%) with lingonberry, blackcurrant, bilberry, raspberry, açai, crowberry, prune or blackberry. *Results*. The groups receiving a high-fat diet supplemented with lingonberries, blackcurrants, raspberries or bilberries gained less weight and had lower fasting insulin levels than the control group receiving high-fat diet without berries. Lingonberries, and also blackcurrants and bilberries, significantly decreased body fat content, hepatic lipid accumulation, and plasma levels of the inflammatory marker PAI-1, as well as mediated positive effects on glucose homeostasis. The group receiving açai displayed increased weight gain and developed large, steatotic livers. Quercetin glycosides were detected in the lingonberry and the blackcurrant diets. *Conclusion*. Lingonberries were shown to fully or partially prevent the detrimental metabolic effects induced by high-fat diet. Blackcurrants and bilberries had similar properties, but to a lower degree. We propose that the beneficial metabolic effects of lingonberries could be useful in preventing obesity and related disorders.

## 1. Introduction

During the last decades, the prevalence of obesity and type 2 diabetes mellitus has increased dramatically. This epidemic of lifestyle-related disorders is affecting all parts of the world, and 439 million people are estimated to suffer from diabetes mellitus in 2030 [1]. Obesity is a strong risk factor for type 2 diabetes with 90% of affected patients being overweight or obese. Obesity is also associated with increased risk of various metabolic disorders including insulin resistance, chronic low-grade inflammation, dyslipidemia, nonalcoholic fatty liver disease (NAFLD), and cardiovascular disease. Oxidative stress, inflammatory response, and altered gut microbiota can play a significant role in the development of obesity-related disorders [2–4]. Type 2 diabetes is a multifactorial disease; however, it appears clear that prevention is possible by avoiding overeating and a sedentary lifestyle to maintain a healthy body weight [5]. The difficulty for many individuals to comply with dietary and lifestyle changes makes it of great interest to identify new foods with well-established effects on preventing the development of obesity and thereby type 2 diabetes and its associated metabolic complications.

Dietary patterns with high consumption of polyphenol rich foods (fruits, vegetables, and berries) have been associated with reduced risk of type 2 diabetes. In general, berries are rich in polyphenols which are suggested to play a role in health benefits of plant-based diets [4, 6–8]. Plant phenolics are a large group of secondary metabolites which provide color and taste in fruits and berries and include flavonoids (anthocyanins, flavonols, and flavanols), tannins, stilbenoids, and phenolic acids [9]. The antioxidant effect of berry anthocyanins has been studied extensively, but still little is known about the biological activities linking berries and polyphenols to the prevention of type 2 diabetes [4, 10, 11].

The aim of the present study was to perform a screening to investigate and compare metabolic effects of different berries in the C57BL/6J mouse. The C57BL/6J is a mouse strain that develops obesity and prediabetes when fed a diet rich in fat, thus mimicking detrimental effects of an energy dense western diet [12]. By supplementing high-fat diets with potentially health-promoting berries, we sought to identify berries capable of ameliorating risk factors from excessive energy intake. Here we report that different berries, possibly due to their polyphenol composition, have varying ability to prevent weight gain and metabolic disorders ultimately leading to diabetes.

## 2. Methods

### 2.1. Animals and Study Design

The study was approved by the Animal Ethics Committee in Lund, Sweden, (Permit Number: M185-11) and is in accordance with the Council of Europe Convention (ETS 123). Male C57BL/6JBomTac mice, 6 weeks old, 21.2 ± 1.1 g were obtained from Taconic (Skensved, Denmark). The animals were housed in a controlled environment (12 h light/dark cycle, light on 7 a.m.). After 9 days of acclimatization the mice were divided into 10 groups of 12 mice each housed in groups of 6 mice per cage. The mice were fed high-fat diets (45 kcal% fat) (Research diets, New Brunswick, NJ, USA) supplemented (20% w/w) with eight different freeze dried berries *ad libitum* for 13 weeks. A control group was fed a macronutrient-matched, isocaloric diet without supplements. One group received a low-fat diet (10 kcal% fat) as an internal control to the high-fat diet induced obesity. Body weight and food intake were monitored weekly throughout the study period. The energy intake was calculated based on registered food consumption. Mice were housed with minimal bedding material and feces was quantitatively collected for two consecutive days at the end of the study and stored at −20°C prior to lyophilization, weighing and powdering with a mortar. At the end of the study, 4 h-fasted animals were anesthetized with an intraperitoneal injection of midazolam (Midazolam Panpharma 5 mg/mL, Panpharma S.A., Luitré, France) and a mixture of fluanisone 10 mg/mL and fentanyl citrate 0.315 mg/mL (Hypnorm, VetaPharma, Leeds, UK). Body composition was determined with dual-energy X-ray absorptiometry (DEXA) technique using a Lunar PIXImus (GE Lunar, Madison, WI, USA). Blood samples were taken by intraorbital puncture. The animals were sacrificed by cervical dislocation and liver, cecum, spleen, and epididymal fat pads were excised, weighed, and snap frozen in liquid nitrogen.

### 2.2. Preparation and Analysis of Diets

The experimental diets were high-fat diets supplemented with one of eight freeze dried berries; Lingonberry (*Vaccinium vitis-idaea*), blackcurrant (*Ribes nigrum*), raspberry (*Rubus idaeus*), bilberry (*Vaccinium myrtillus*), and blackberry (*Rubus fruticosus*) were obtained from MOLDA AG (Dahlenburg, Germany). Crowberries (*Empetrum nigrum*) were from Olle Svenssons Partiaffär AB (Olofström, Sweden) and prunes (*Prunus domestica*) from Semper AB (Sundbyberg, Sweden). Freeze dried açai berry powder (*Euterpe oleracea*) was purchased from Superfruit Scandinavia AB (Sweden). Information about origin and processing of the berries can be found in Table S2 (see Supplementary Material available online at http://dx.doi.org/10.1155/2014/403041). Based on data of macronutrient composition of the berries (obtained from supplier and/or Covance, Madison, WI, USA) the diets were designed to have identical macronutrient composition (Tables  1 and S1, Supporting information). After manufacturing, all diets were analyzed for soluble and insoluble dietary fiber content by Eurofins (Lidköping, Sweden) (Table 1).

### 2.3. Analysis of Plasma Samples and Assessment of Insulin Resistance

Plasma was prepared by immediate centrifugation of blood samples. Glucose, total cholesterol, triacylglycerol, high-density lipoprotein (HDL) cholesterol, alanine aminotransferase (ALT) (Infinity, Thermo Fisher Scientific, Waltham, MA, USA), and nonesterified fatty acid (NEFA) (NEFA-HR, Wako Chemicals, Neuss, Germany) concentrations in plasma were measured using kits. Low-density lipoprotein (LDL) cholesterol was estimated by the Friedewald formula [13]. Insulin was measured using an enzyme-linked immunosorbent assay kit (Mercodia, Uppsala, Sweden). Plasma levels of tumor necrosis factor-alpha (TNF-*α*) and plasminogen activator inhibitor-1 (PAI-1) were analyzed using Luminex technology (LX200, Luminex Corporation, Austin, TX, USA). Insulin resistance was assessed by the homeostasis model assessment (HOMA), a mathematical model describing the degree of insulin resistance from fasting plasma glucose and insulin [14, 15]. Homeostasis model assessment-estimated insulin resistance (HOMA-IR) was calculated by multiplying fasting plasma insulin (mU/L) with fasting plasma glucose (mmol/L) divided by 22.5.

### 2.4. Fecal Analyses

Lipids were extracted from feces using a modified version of the protocol by Hara and Radin [16]. In short, around 100 mg lyophilized, grounded feces from each sample were extracted in hexane-isopropanol (3 : 2 v/v) with 0,005% 2,6-di-tert-butyl-4-methylphenol. Five mL of the extract was dried under N_2_ after which the remaining residue was redissolved in 100 *μ*L isopropanol containing 1% Triton X-100. The solution was analyzed in triplicates using the triacylglycerol and cholesterol kits used for plasma samples.

### 2.5. Extraction and Quantification of Liver Lipids

The frozen livers were grounded to a powder in a mortar under liquid nitrogen. Samples were prepared in duplicates from ten randomly selected livers from each group and subjected to lipid extraction as previously described. For extraction of hepatic lipids, 20 mg of frozen liver powder was used. The solution containing redissolved lipids was first analyzed in triplicates for cholesterol. After adding another 90 *μ*L isopropanol 1% Triton X-100 the solution was analyzed for triacylglycerol.

### 2.6. Extraction of Polyphenols from Diets

Five g of each diet was grounded, weighed, and extracted using 25 mL of heptane, ethyl acetate, and methanol, respectively. Samples were stirred during 24 h at room temperature and the extracts were filtered, concentrated, and weighed. Finally, 25 mL of methanol : water : acetic acid (85 : 15 : 0.5; v/v) was used for polyphenol analysis. The extraction was carried out using 15 min of sonication. Ethyl acetate extracts were dissolved in 2 mL methanol and the soluble fraction was filtered through 0.2 *μ*m GH polypro membrane to remove insoluble particles and kept at −18°C until analysis.

### 2.7. LC-MS Analysis of Polar Polyphenols (Anthocyanins)

The liquid chromatography-tandem mass spectrometry (LC-MS) analyses were performed on a LC-MS Agilent technologies 1260 infinity equipped with an quaternary pump, autosampler, thermostatted column compartment, diode array detector tandem quadrupole LC/MS. A 250 × 4.6 mm i.d. Zorbax SB-C_18_ column, 3.5 *μ*m particle size, was used at 40°C. The method used is described by Wu et al. [17] using mobile phase A (5% formic acid in water) and B (methanol). The flow rate was 1 mL/min, the temperature used was 40°C, and UV detection was at 520 nm. The gradient used was 5% B, 0–2 min; 5–20% B, 2–10 min; 20% B, 10–15 min; 20–30% B, 15–30 min; 30% B, 30–35 min; 30–45% B, 35–50 min; 45% B, 50–55 min, 45–5% B 55–65 min, 5% B. The injection volume was 20 *μ*L in all samples. Enhanced product ion mass spectrometry (EPI-MS) analysis was performed in positive mode using a capillary voltage of 3000 V, nebulizer pressure 40 psig, drying gas flow 12 L/min, and drying gas temperature 300°C. The UV-vis, reference times, and mass spectra were used for identification of the peaks and compared to anthocyanin data in the literature [17–19].

### 2.8. LC-MS Analysis of Medium Polar Polyphenols

The LC-MS system together with a 2.1 × 100 mm i.d. 3 *μ*m Atlantis C18 column was used for the analysis of the mobile phase A (0.1% formic acid in water) and mobile phase B (methanol). The flow rate was 0.3 mL/min, the temperature used was 30°C, and the UV detector was fixed at 360 nm. The injection volume was 20 *μ*L. The initial gradient elution was used at 6–12% B, 20 min; 12–55% B, 20–50 min. The conditions for MS were set in both positive and negative mode (method described in [20]). Qualitative standards (resveratrol, apigenin, and quercetin-3-O-glucoside) as well as comparison of MS data in the literature were used to enable identification of compounds.

### 2.9. Statistical Analysis

Data are presented as mean ± standard error of the mean (SEM). Unless stated otherwise, results were analyzed by one-way analysis of variance (ANOVA) in conjunction with Dunnett's multiple comparisons test. In cases where Gaussian distribution could not be assumed, groups were compared using Kruskal-Wallis and Dunn's post test. All results are compared to the high-fat control group. Differences with a *P* value < 0.05 were considered significant. *P < 0.05, **P < 0.01 and ***P < 0.001. Statistical analyses were performed using GraphPad Prism versions 5.0 and 6.0 (GraphPad Software, San Diego, CA, USA).

## 3. Results

### 3.1. Energy Intake, Body Weight, and Body Fat Content

**Figure 1:**
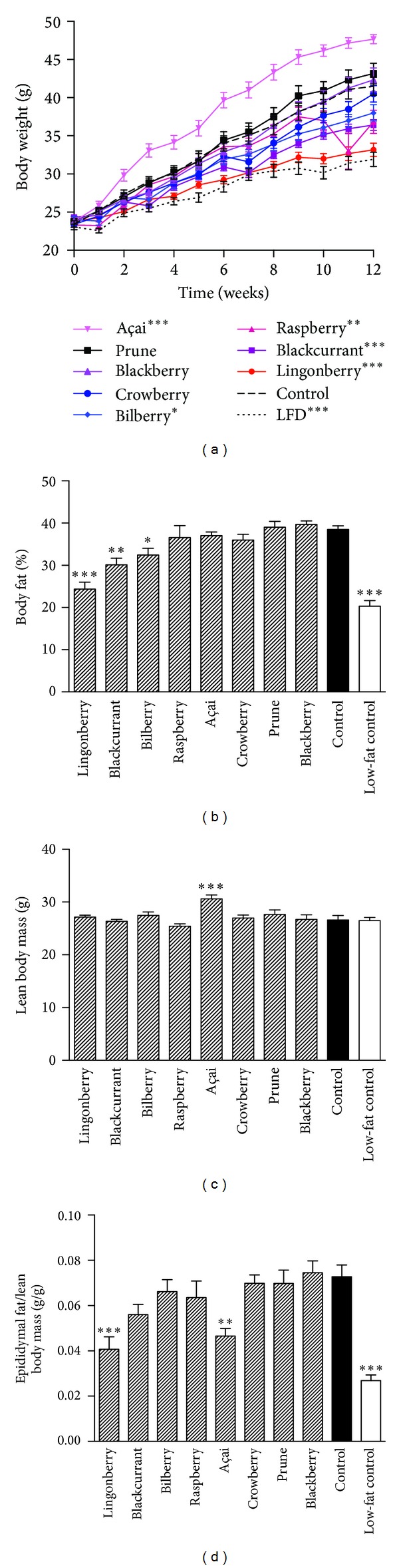
Body weight and composition after 13 weeks of high-fat diet and berry supplementation. (a) Weekly body weight registration. Statistical comparisons of body weight compared to control were made using a two-way ANOVA with Bonferroni post test. The stars represent significant differences in body weight at the last time point of weight registration before ending the study. Body fat (b) and lean body mass (c) were recorded using DEXAscan technique at week 13 of the study. (d) Epididymal white adipose tissue weight related to lean body mass after 13 weeks on the different diets (*n* = 11-12). Values represent mean ± SEM, *n* = 12 mice/group. Mean values significantly different from the control are denoted with *P < 0.05, **P < 0.01 or ***P < 0.001.

The high-fat control diet induced obesity in mice was compared to the low-fat diet group (Figure 1(a)). In week 12 of the experiment, the mean body weight in the low-fat group was 32 ± 0.9 g, whereas the mean weight of the high-fat fed control group was 42 ± 1.2 g. Body weight was significantly lower in groups receiving lingonberry (33 ± 0.9 g), blackcurrant (36 ± 0.7 g), raspberry (37 ± 1.5 g), and bilberry (38 ± 1.1 g) compared to the high-fat control. Consumption of the açai diet resulted in significantly increased body weight (48 ± 0.6 g). The DEXA scan showed significantly decreased body fat content in the groups receiving lingonberry, blackcurrant, and bilberry compared to the high-fat control. In fact, mice fed the lingonberry-supplemented diet had a body fat content as low as the low-fat diet group. The lean body mass was similar in all groups (Figure 1(c)), except in the group receiving açai where lean mass was increased (+15%) compared to the control. The mass of the epididymal fat pads was expressed per gram lean tissue to take into account differences in body size. The relative size of the fat pad was lower in groups receiving lingonberry and açai, compared to the high-fat control (Figure 1(d)). The accumulated mean energy intake per body weight was similar for all groups, except for mice receiving blackcurrant and bilberry supplementation where a higher food and energy intake was registered (Figure S1).

### 3.2. Plasma Parameters and Insulin Resistance Index

**Figure 2:**
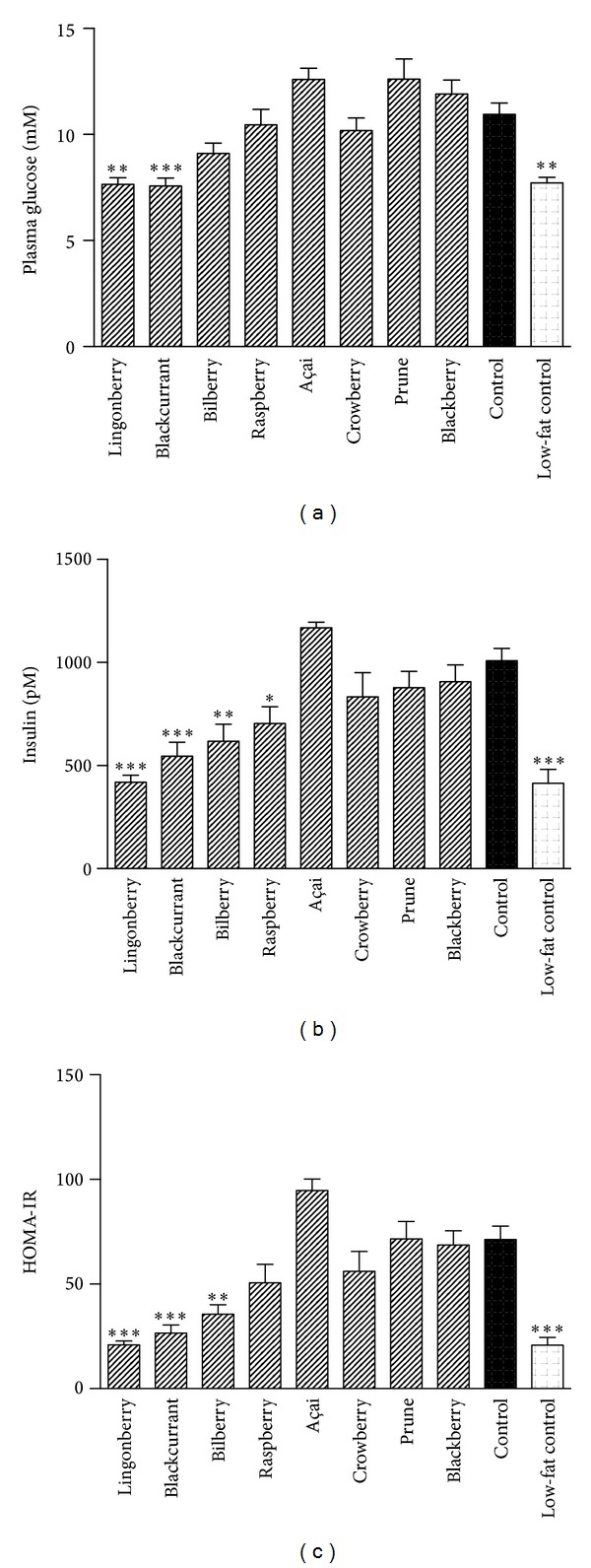
Glycemic control in mice after 13 weeks of high-fat diet and berry supplementation. Plasma glucose (a) and insulin (b) concentrations after 4-hour fasting were used to calculate HOMA-IR index (c). Results represent mean ± SEM, *n* = 11-12. Values significantly different from the control are denoted: *P < 0.05, **P < 0.01, ***P < 0.001.

Four-hour fasting plasma glucose levels were significantly reduced in groups receiving lingonberry- and blackcurrant-supplemented diets compared to the high-fat control diet (Figure 2(a)). These groups together with the bilberry and raspberry groups had reduced fasting insulin levels (Figure 2(b)). In addition, the lingonberry, blackcurrant, and bilberry supplementation resulted in a lower HOMA index of insulin resistance (Figure 2(c)). The lingonberry and blackcurrant groups had glucose, insulin, and insulin resistance levels very similar to the group receiving a low-fat diet. A tendency of increased HOMA-IR, glucose, and insulin was observed in mice consuming açai-supplemented high-fat diet compared to control; however, the increase was not significant (*P* value; 0.07, 0.25 and 0.55, resp.).

The plasma lipid profiles are shown in Table 2. Compared to control, the total plasma cholesterol was significantly lower in groups fed lingonberry, blackcurrant, and low-fat diet whereas it tended to be higher in the acai group (*P* = 0.05). The lingonberry, blackcurrant, and low-fat groups displayed decreased levels of LDL and HDL cholesterol whereas açai had increased HDL cholesterol. However, there were no significant changes regarding the calculated LDL/HDL ratio. Plasma triacylglycerol levels were significantly increased in the group receiving blackcurrant and in the low-fat control. There were no significant differences in circulating nonesterified fatty acids.

### 3.3. Liver Lipid Accumulation and Liver Function

**Figure 3:**
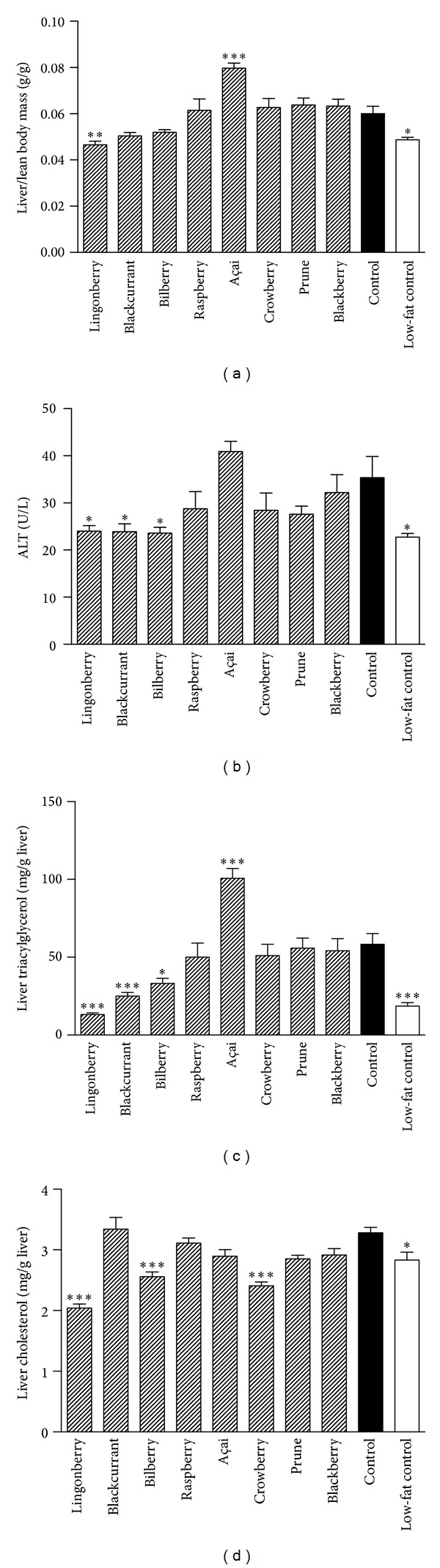
Effects of berry supplementation on liver size, function and lipid accumulation. (a) Liver weights, expressed as gram per gram lean body mass, after 13 weeks of high-fat diet supplemented with different berries. (b) Plasma alanine aminotransferase (ALT) concentration (units per liter). Liver triacylglycerol (c) and cholesterol (d) content, expressed as mg per g liver weight. Data are means ± SEM, *n* = 10–12. *P < 0.05, **P < 0.01, ***P < 0.001.

The liver masses (relative to lean body mass) were significantly lower in mice fed a diet supplemented with lingonberries compared to control (*P* = 0.009), whereas açai supplementation led to significantly increased liver mass (*P* < 0.0001) (Figure 3(a)). The plasma levels of the enzyme ALT, a marker of liver dysfunction, were significantly elevated in the açai group compared to all groups except the blackberry and control groups (Figure 3(b)). Compared to the high-fat control, ALT levels were significantly reduced in groups receiving lingonberry and blackcurrant as well as bilberry. The liver contents of triacylglycerol (Figure 3(c)) were markedly decreased in the mice receiving supplement of lingonberries, blackcurrant, and, to a lower degree, bilberries whereas açai induced an increase in liver triacylglycerol. Lingonberries, bilberries, and crowberries diets reduced liver cholesterol content compared to the high-fat control (Figure 3(d)). All the studied liver parameters were decreased in the low-fat diet compared to the high-fat control.

### 3.4. Effect of Berry Supplementation on Fecal Excretion and Cecal Weight

The total amount of feces collected over 24 h as well as fecal excretion of triacylglycerol and cholesterol is presented in Table 3. There were no significant differences in total amount of excreted feces, whereas fecal content of cholesterol was elevated in the bilberry, açai, crowberry, blackberry, and low-fat control group. Compared to control, the amount of excreted triacylglycerol was significantly higher in all groups except the low-fat control and the group receiving prune. The first part of the large intestine (cecum) is a site for bacterial fermentation. The mass of the cecum, including content, was increased in all groups compared to control except in the raspberry group (Table 3). In this study, bilberry and lingonberry-supplemented diets gave rise to the largest cecum masses.

### 3.5. Effects of Berry Supplementation on Inflammation

**Figure 4:**
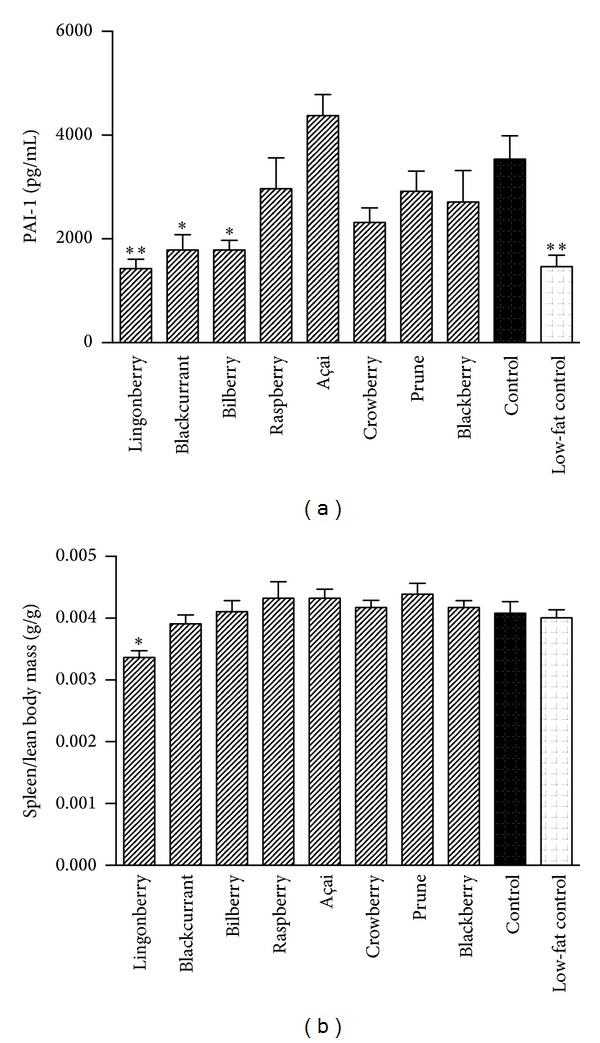
Effect of berry supplementation on inflammatory markers. (a) The plasma concentration of PAI-1 (plasminogen activator inhibitor 1), mirroring low-grade inflammation, was reduced in mice receiving lingonberries, blackcurrants, and bilberries compared to control. Spleen weight (b) related to lean body mass was reduced in the lingonberry group compared to the control. Data are means ± SEM, *n* = 10–12. *P < 0.05, **P < 0.01.

In order to assess the effects of berry supplementation on low-grade inflammation, plasma levels of the inflammation markers PAI-1 (Figure 4(a)) and TNF-*α* were measured. The PAI-1 concentration was lower in plasma from mice receiving lingonberries, blackcurrants, bilberries, and low-fat diet compared to control. The PAI-1 levels in mice receiving açai were elevated compared to other groups, although not significantly compared to control. Plasma TNF-*α* was below the detection limit in all samples. Spleen size is sometimes analyzed as a reflection of inflammatory activity [21]. In this study, the spleen mass relative to lean tissue mass was significantly lower in the lingonberry group compared to high-fat control (Figure 4(b)).

### 3.6. Polyphenolic Compounds in Berry Diets

The polyphenols detected in the different berry diets are displayed in Table 4. Anthocyanins were present in extracts from all experimental diets, except the prune diet. However, medium polar polyphenols were only detected in the lingonberry and blackcurrant diets. Quercetin-3-O-glucoside and quercetin-3-O-galactoside were identified in both lingonberry and blackcurrant diets and in agreement with the earlier literature [20] quercetin-3-O-*α*-rhamnoside (quercitrin), kaempferol-deoxyhexoside, and quercetin-3-O-(4′′-HMG)-*α*-rhamnoside were identified in the lingonberry diet. Resveratrol and apigenin were not detected in our analysis. No polyphenol signal was detected in the control diets.

## 4. Discussion

In this study, we show that intake of certain species of berries can prevent weight gain and counteract the metabolic derangements induced by a high-fat diet. Dietary intake of lingonberries prevented adiposity, hepatic lipid accumulation, alleviated hyperglycemia, hyperinsulinemia, and dyslipidemia and decreased plasma PAI-1 and ALT levels in mice fed a high-fat diet. Blackcurrants and, to a lower extent, bilberries and raspberries had similar effects, whereas neither crowberries, prunes, nor blackberries caused any significant improvements of metabolic parameters in this study.

The almost complete prevention of body weight gain observed in the group receiving lingonberry-supplemented diet is an effect of reduced adiposity. Our finding is in agreement with a study in Wistar rats, in which it was shown that lingonberry extracts favorably affected antioxidant defense enzymes, but it was also apparent that the lingonberry extracts reduced weight gain compared to the control [22]. However, to the best of our knowledge, there are no other publications addressing the antiobesity effect of lingonberries. Increased fat excretion is unlikely to entirely explain the protection against adiposity since fecal excretion of triacylglycerol was not elevated in the lingonberry group compared to groups receiving some of the other berries. Also, there was no correlation between body weight and dietary intake of soluble, insoluble, or total fiber (data not shown). Interestingly, blackcurrant supplementation efficiently prevented weight gain and body fat accumulation despite an increased energy intake. However, the monitoring of caloric intake was based on registered food intake and due to texture, the blackcurrant diet gave rise to a higher spillage than other diets which could be incorrectly interpreted as a higher food intake. The bilberry group also had a higher food intake compared to control. The increased excretion of triacylglycerol in bilberries, blackcurrants, and raspberries could be caused by reduced energy absorption and explain some of the beneficial effects on adiposity. This potential mechanism should be further investigated since increased fat excretion also was observed by supplementation with crowberry, acai, and blackberries, without preventing weight gain. Polyphenol-rich extracts have been shown to inhibit pancreatic lipase [23, 24] although it remains to be established if this mechanism operates also in vivo.

Approximately 70–80% of patients with type 2 diabetes suffer from nonalcoholic fatty liver disease (NAFLD), which is linked to increased risk of cardiovascular disease [25, 26]. Fat accumulation in the liver is associated with impaired hepatic insulin sensitivity [27–29] and production of inflammatory markers [30, 31]. Interestingly, lingonberry supplementation significantly reduced liver mass and liver lipid accumulation. Elevated ALT levels in plasma correlate with reduced liver function and steatosis and predicts type 2 diabetes [32–34]. In this study, the significant reduction in liver triacylglycerol and ALT levels in the lingonberry, blackcurrant, and bilberry groups implies protection against liver steatosis and subsequent improved liver function. Our findings are supported by a 20-week dietary intervention study where a diet rich in berries (bilberries, sea buckthorns, blackcurrants, and lingonberries) reduced ALT in overweight women [35]. However, in a follow-up study using only bilberries and sea buckthorn no effects on ALT-levels were observed; thus, the authors concluded that either the lingonberries and/or blackcurrants were responsible for modulating hepatic lipid metabolism in a positive direction with lower ALT as a result [36]. In contrast to the beneficial effects observed following intake of lingonberry, blackcurrant, or bilberry, livers dissected from mice receiving supplementation with açai were large and visibly more whitish than other groups, indicating liver steatosis. Analysis revealed that the livers weighed more and had higher triacylglycerol content, and mice receiving açai had significantly higher plasma ALT levels than the rest of the berry groups, but not compared to the control group. Taken together, this suggests that açai promotes rather than prevents fatty liver. However, lipid accumulation per se may not be causal for the insulin resistance associated with fatty liver [37].

Mice fed lingonberries, blackcurrants, and bilberries had lower fasting glucose and/or insulin, resulting in a lower HOMA-insulin resistance index (Figure 2). Thus our data indicate that these three berries protected against high-fat induced insulin resistance. The improved glucose control may be an effect of the reduced adiposity. However, in humans, ingestion of lingonberries has been shown to abolish and decrease, respectively, the postprandial hyperglycemic response of ingesting glucose or sucrose in normal-weight healthy subjects [38, 39]. A cell-assay based bioactivity screening of plants traditionally used to treat symptoms related to diabetes in a native Canadian population identified an extract of lingonberries as having the highest antidiabetic potential [40]. A follow-up study by Eid et al. revealed that a lingonberry extract stimulated glucose uptake into muscle cells by activating the AMP-activated protein kinase (AMPK) pathway [41]. Also, a bilberry extract fed to diabetic KK-A^*y*^ mice ameliorated hyperglycemia and enhanced insulin sensitivity accompanied by AMPK activation [10].

In the polyphenolic characterization of the diets, quercetin-3-O-glucoside and quercetin-3-O-galactoside were found in the lingonberry and the blackcurrant diets. Lingonberries and bilberries belong to the *Vaccinium *genus and Eid et al. proposed that the traditional use of this genus to treat diabetes is due to the ability of quercetin and some of its glucosides to transiently inhibit ATP synthase and activate AMPK [41]. In a study by Kobori et al., supplementation with 0.05% quercetin alleviated hepatic fat accumulation and decreased visceral fat deposition, hyperglycemia, hyperinsulinemia, dyslipidemia, and inflammation in C57BL/6J mice fed a high-fat Western diet [42]. Since lingonberries and blackcurrants had the most beneficial effects in our study, the finding that quercetin glycosides were detected only in the lingonberry and blackcurrant diets is interesting and will be further evaluated. Moreover, Erlund et al. have shown that daily intake of lingonberries, blackcurrants, and bilberries significantly increase, serum quercetin levels in humans [43].

Berries are in general rich in anthocyanins and several anthocyanins were found in the experimental diets. Many of the detected anthocyanins and quercetin glycosides are associated with relevant bioactivities [11, 42, 44, 45], but our study design does not permit elaboration of causal relationships between specific compounds and health outcomes. The fact that resveratrol was not detected in the diets is somewhat unexpected since its presence in some of the studied berries has been demonstrated [46]. However, contents of polyphenols are known to vary considerably due to different varieties, cultivars, and growing conditions [47].

Obesity and type 2 diabetes are associated with a systemic low-grade inflammation, and it has been suggested that an altered gut microbiota could be the underlying cause of this inflammation [48–50]. Berries are high in fiber and phenolic compounds which could promote or inhibit growth of certain species of bacteria and thereby alter the microbiota. The finding that all berry diets, except raspberries, increased cecum mass suggests a change in microbial fermentative activity. Dietary administration of lingonberries, blackcurrants, and bilberries reduced plasma levels of PAI-1, which indicate an anti-inflammatory effect. In addition, the significantly lower spleen mass observed in the lingonberry-group may reflect a reduced systemic inflammation. Many previous studies on berries have focused on the capacity of antioxidative polyphenols to reduce oxidative stress and prevent cardiovascular disease. Increased PAI-1 concentrations result in reduced fibrinolytic activity and play a key role in atherothrombosis [51]. Elevated PAI-1 is also associated with NAFLD [52]. The reduction of PAI-1 in combination with lower plasma cholesterol levels by lingonberry and blackcurrant supplementation suggests that these berries may be useful in preventing cardiovascular events. However, there was no significant effect on LDL/HDL cholesterol ratio and in the blackcurrant group an increase in plasma triacylglycerol was observed. In contrast, mice receiving açai had increased total cholesterol and PAI-1 in plasma, further questioning the health aspects of this berry.

In conclusion, this study demonstrated that daily supplementation with lingonberries and also blackcurrants and bilberries had pronounced antiobesity and beneficial metabolic effects in high-fat fed C57BL/6J mice. The mechanisms behind the effects should be further evaluated, taking into account lower doses and reproducibility in humans. The capacity of lingonberries to counteract the negative outcomes of an unhealthy diet could be useful in designing dietary intervention strategies aimed at preventing development of obesity and type 2 diabetes.

## Supplementary Material

Table S1. Formulation of diets. The diets are formulated to have matched macronutrient composition by energy. Diets manufactured by Research diets, NB, USA.
Table S2. Origin and processing of berries. Dried berries were obtained from various sources before being sent to Research diets, NB, USA and incorporated into mouse diets.
Figure S1. Cumulative food intake during 13 weeks of high-fat diet and berry supplementation. Weekly assessment of food intake in each cage (2 cages per group, 6 mice per cage) show increased consumption in the groups receiving blackcurrant and bilberry.

## Figures and Tables

**Table 1:** Composition of diets^1^.

	Lingonberry	Blackcurrant	Bilberry	Raspberry	Açai	Crowberry	Prune	Blackberry	Control	LFD
*Calculated energy (kcal) *										
Protein	812.0	812.0	812.0	812.0	812.0	812.0	812.0	812.0	812.0	812.0
Carbohydrate	1422.4	1422.4	1422.4	1422.4	1422.4	1422.4	1422.4	1422.4	1422.4	2840.4
Starch	731.2	731.2	731.2	731.2	731.2	731.2	731.2	731.2	731.2	2149.2
Sucrose	347.2	347.2	347.2	347.2	347.2	347.2	347.2	347.2	347.2	347.2
Fructose	172.0	172.0	172.0	172.0	172.0	172.0	172.0	172.0	172.0	172.0
Glucose	172.0	172.0	172.0	172.0	172.0	172.0	172.0	172.0	172.0	172.0
Fat	1822.5	1822.5	1822.5	1822.5	1822.5	1822.5	1822.5	1822.5	1822.5	405.0
Fiber	0.0	0.0	0.0	0.0	0.0	0.0	0.0	0.0	0.0	0.0
Other	0.0	0.0	0.0	0.0	0.0	0.0	0.0	0.0	0.0	0.0
Total kcals	4057	4057	4057	4057	4057	4057	4057	4057	4057	4057
*Calculated energy per gram diet (kcal/g) *										
kcal/g	4.2	4.1	4.3	4.3	4.2	4.2	4.6	4.5	4.5	3.7
*Calculated energy (kcal%) *										
Protein	20	20	20	20	20	20	20	20	20	20
Carbohydrate	35	35	35	35	35	35	35	35	35	70
Fat	45	45	45	45	45	45	45	45	45	10
Fiber	0	0	0	0	0	0	0	0	0	0
*Analyzed fiber^2^* * (g/100 g diet) *										
Insoluble fiber	7.4	8.2	9.3	10.8	8.8	12.5	6.3	11.9	10.1	8.5
Soluble fiber	1.8	2.5	<1	1.2	1.6	<1	1.1	<1	<1	<1
Total fiber	9.2	10.7	10.0	12.0	10.4	13.0	7.4	12.6	10.5	9.1

^1^All diets were designed to have an equal caloric content of fat, protein, and carbohydrates (including glucose, fructose, and sucrose). LFD: low-fat diet.

^2^Fiber analyzed by Eurofins, Sweden.

**Table 2:** Plasma lipid profiles (mM) in mice after 13 weeks on high-fat diets supplemented with berries^1^.

	Triacylglycerol		Total cholesterol		HDL cholesterol		LDL cholesterol		LDL/HDL ratio		NEFA	
	Mean	SEM	Mean	SEM	Mean	SEM	Mean	SEM	Mean	SEM	Mean	SEM
Lingonberry	0.65	0.06	2.6***	0.11	1.2***	0.04	1.0**	0.06	0.84	0.04	0.56	0.04
Blackcurrant	0.83*	0.04	2.9**	0.14	1.4**	0.06	1.1*	0.09	0.76	0.06	0.66	0.03
Bilberry	0.59	0.06	3.4	0.15	1.7	0.06	1.5	0.08	0.87	0.03	0.57	0.06
Raspberry	0.66	0.03	3.6	0.24	1.8	0.13	1.5	0.14	0.87	0.05	0.54	0.04
Açai	0.59	0.03	4.3	0.13	2.1*	0.08	1.9	0.07	0.90	0.04	0.49	0.03
Crowberry	0.56	0.04	4.1	0.11	2.0	0.05	1.8	0.08	0.91	0.04	0.54	0.05
Prune	0.57	0.03	4.1	0.15	2.0	0.06	1.8	0.15	0.94	0.08	0.45	0.02
Blackberry	0.67	0.02	4.0	0.17	1.9	0.08	1.8	0.13	0.92	0.06	0.60	0.04
Control	0.64	0.03	3.7	0.16	1.8	0.07	1.6	0.11	0.88	0.05	0.56	0.05
Low-fat control	0.84*	0.09	2.4***	0.15	1.1***	0.06	0.85***	0.13	0.76	0.12	0.67	0.05

^1^Mean values significantly different from control are depicted with *P < 0.05, **P < 0.01, respectively, ***P < 0.001. SEM: standard error of the mean. *n* = 12 mice from all groups except *n* = 11 in the lingonberry group in low density-lipoprotein LDL and low-/high-density lipoprotein LDL/HDL cholesterol. NEFA: nonesterified fatty acids.

**Table 3:** Effect of berry supplementation on cecum weight, total fecal excretion, and lipid excretion^1^.

	Cecum weight (g)		Dry feces (g/c/24 h)		Fecal triacylglycerol (g/c/24h)		Fecal cholesterol (g/c/24 h)	
	Mean (*n* = 12)	SEM	Mean (*n* = 4^†^)	SD	Mean (*n* = 4^†^)	SD	Mean (*n* = 4^†^)	SD
Lingonberry	0.64***	0.028	2.44	0.15	6.22***	1.22	3.74	0.93
Blackcurrant	0.35**	0.022	2.12	0.45	10.68***	3.45	2.80	0.39
Bilberry	0.78***	0.035	2.86	0.25	15.18***	1.61	5.88***	1.21
Raspberry	0.29	0.013	2.85	0.40	7.89***	1.36	3.74	0.34
Açai	0.50***	0.031	3.18	0.55	5.57**	1.64	5.75***	1.03
Crowberry	0.42***	0.030	3.41	0.21	9.50***	1.83	7.52***	0.93
Prune	0.38***	0.023	2.25	0.12	0.96	0.26	3.91	0.53
Blackberry	0.35**	0.019	3.15	0.34	4.73**	1.16	2.33*	0.41
Control	0.21	0.012	2.85	0.24	1.40	0.55	3.60	0.21
Low-fat control	0.32*	0.036	2.68	0.30	0.77	0.14	2.26*	0.15

^1^Statistical comparisons of cecum weight were made using ANOVA and Dunnett's posttest, all groups compared to control, *P < 0.05, **P < 0.01, ***P < 0.001. Remaining statistical comparisons to control were made using repeated-measures two-way ANOVA and Sidak's posttest. SEM: standard error of the mean; SD: standard deviation. ^†^The number refers to the number of observations (two cages (c) per group analyzed over 24 hours, two days in a row). Six mice were housed in each cage.

**Table 4:** Polyphenolic composition of berry diets.

Anthocyanins	Lingonberry	Blackcurrant	Bilberry	Raspberry	Açai	Crowberry	Prune	Blackberry
Cyanidin-3-glucoside	x	x	x	x	x	x		x
Cyanidin-3-galactoside	x		x			x		
Cyanidin-3-arabinoside	x		x			x		
Cyanidin-3-sophoroside				x				
Cyanidin-3-rutinoside				x	x			
Cyanidin-3- (6′′-malonyl)-glucoside						x		x
Delphinidin-3-rutinoside		x		x				
Delphinidin-3-glucoside			x			x		
Delphinidin-3-galactoside			x			x		
Delphinidin-3-arabinoside			x			x		
Petunidin-3-rutinoside				x				
Petunidin-3-galactoside			x			x		
Petunidin-3-glucoside			x					
Petunidin-3-arabinoside			x			x		
Peonidin-3-rutinoside				x	x			
Peonidin-3-galactoside			x			x		
Peonidin-3-glucoside			x					
Peonidin-3-arabinoside						x		
Malvidin-3-galactoside			x			x		
Malvidin-3-glucoside			x					
Malvidin-3-arabinoside			x			x		
*Medium polar polyphenols *								
Quercetin-3-O-glucoside	x	x						
Quercetin-3-O-galactoside	x	x						
Quercetin-3-O-*α*-rhamnoside	x							
Kaempferol-deoxyhexoside	x							
Quercetin-3-O-(4′′-HMG)-*α*-rhamnoside	x							
