# Supplementary material for: Evaluation of Beneficial Metabolic Effects of Berries in High-Fat Fed C57BL/6J Mice
Source: J Nutr Metab. 2014 Jan 14;2014:403041. doi: 10.1155/2014/403041 (PMC3941780; doi:10.1155/2014/403041)
Supplement: Supplementary file 1 — Table S1. Formulation of diets. The diets are formulated to have matched macronutrient composition by energy. Diets manufactured by Research diets, NB, USA. Table S2. Origin and processing of berries. Dried berries were obtained from various sources before being sent to Research diets, NB, USA and incorporated into mouse diets. Figure S1. Cumulative food intake during 13 weeks of high-fat diet and berry supplementation. Weekly assessment of food intake in each cage (2 cages per group, 6 mice per cage) show increased consumption in the groups receiving blackcurrant and bilberry. [file 403041.f1.pdf]

Table S1. Formulation of diets

	Low-Fat Diet (D11030901)	High-Fat Control (D11030902)	Bilberry (D11030903)	Raspberry (D11030904)	Blackberry (D11030905)	Blackcurrant (D11030906)	Lingonberry (D11030907)	Crowberry (D11030908)	Prune (D11030909)	Açai (D11030910)
**Ingredient (g/kg)**										
Casein	182.3	222.2	200.2	188.7	196.2	191.5	207.3	203.4	218.3	201.5
L-Cystine	2.7	3.3	3.1	3.1	3.2	3.1	3.2	3.1	3.4	3.4
Corn starch	407.7	91.3	85.4	84.2	87.5	84.8	85.8	58.3	11.1	68.8
Maltodextrin 10	82.0	111.1	103.9	101.7	105.7	102.4	105.8	104.3	112.2	111.8
Sucrose	70.0	85.3	78.8	55.3	80.4	71.7	77.7	79.8	83.7	85.9
Fructose	39.2	47.8	0.3	25.3	4.1	11.7	0.1	17.8	20.0	44.3
Dextrose (Glucose)	39.2	47.8	13.9	30.5	8.5	20.1	0.5	16.4	8.2	43.5
Cellulose, BW200	83.8	102.1	51.9	50.8	52.9	51.2	52.9	52.1	56.1	55.9
Soybean Oil	22.8	27.8	26.0	25.4	26.4	25.6	26.5	26.1	28.0	28.0
Lard	18.2	197.2	176.8	177.1	174.9	179.6	179.9	179.1	195.1	93.3
Minerals Mix S10026	9.1	11.1	10.4	10.2	10.6	10.2	10.6	10.4	11.2	11.2
DiCalcium Phosphate	11.9	14.4	13.5	13.2	13.7	13.3	13.8	13.6	14.6	14.5
Calcium Carbonate	5.0	6.1	5.7	5.6	5.8	5.6	5.8	5.7	6.2	6.2
Potassium Citrate, 1 H2O	15.0	18.3	17.1	16.8	17.4	16.9	17.5	17.2	18.5	18.5
Vitamin Mix V10001	9.1	11.1	10.4	10.2	10.6	10.2	10.6	10.4	11.2	11.2
Choline Bitartrate	1.8	2.2	2.1	2.0	2.1	2.0	2.1	2.1	2.2	2.2
Bilberry	0.0	0.0	200.0	0.0	0.0	0.0	0.0	0.0	0.0	0.0
Raspberry	0.0	0.0	0.0	200.0	0.0	0.0	0.0	0.0	0.0	0.0
Blackberry	0.0	0.0	0.0	0.0	200.0	0.0	0.0	0.0	0.0	0.0
Blackcurrant	0.0	0.0	0.0	0.0	0.0	200.0	0.0	0.0	0.0	0.0
Lingonberry	0.0	0.0	0.0	0.0	0.0	0.0	200.0	0.0	0.0	0.0
Crowberry	0.0	0.0	0.0	0.0	0.0	0.0	0.0	200.0	0.0	0.0
Prune	0.0	0.0	0.0	0.0	0.0	0.0	0.0	0.0	200.0	0.0
Açai	0.0	0.0	0.0	0.0	0.0	0.0	0.0	0.0	0.0	200.0
**Total**	1000.0	1000.0	1000.0	1000.0	1000.0	1000.0	1000.0	1000.0	1000.0	1000.0

The diets are formulated to have matched macronutrient composition by energy. Diets manufactured by Research diets, NB, USA.

Table S2. Origin and processing of berries

	Lingonberry	Blackcurrant	Bilberry	Raspberry	Açai	Crowberry	Prune	Blackberry
**Scientific name**	*Vaccinium vitis-idaea*	*Ribes nigrum*	*Vaccinium myrtillus*	*Rubus idaeus*	*Euterpe Oleraceae*	*Empetrum nigrum*	*Prunus domestica*	*Rubus fruticosus*
**Supplier /manufacturer**	Procordia Food (Eslöv, Sweden) /MOLDA (Germany)	Procordia Food /MOLDA	Procordia Food /MOLDA	Procordia Food /MOLDA	Superfruit (Sweden)	Procordia Food /Olle Svenssons Partiaffär (Sweden)	SEMPER (Sundbyberg, Sweden)	Procordia Food /MOLDA
**Origin**	Europe, Sweden	Eastern Europe, Serbia, Montenegro	Russia, Poland, Ukraine, Scandinavia	Poland, Chile, Serbia and Bulgaria	Brazil	Finland	France	Eastern Europe, Serbia, Montenegro
**Processing**	Freeze-dried	Freeze-dried	Freeze-dried	Freeze-dried	Citric acid added to pasteurized acai pulp. Freeze-dried.	Freeze-dried	Roller-dried	Freeze-dried

Dried berries were obtained from various sources before being sent to Research diets, NB, USA and incorporated into mouse diets.

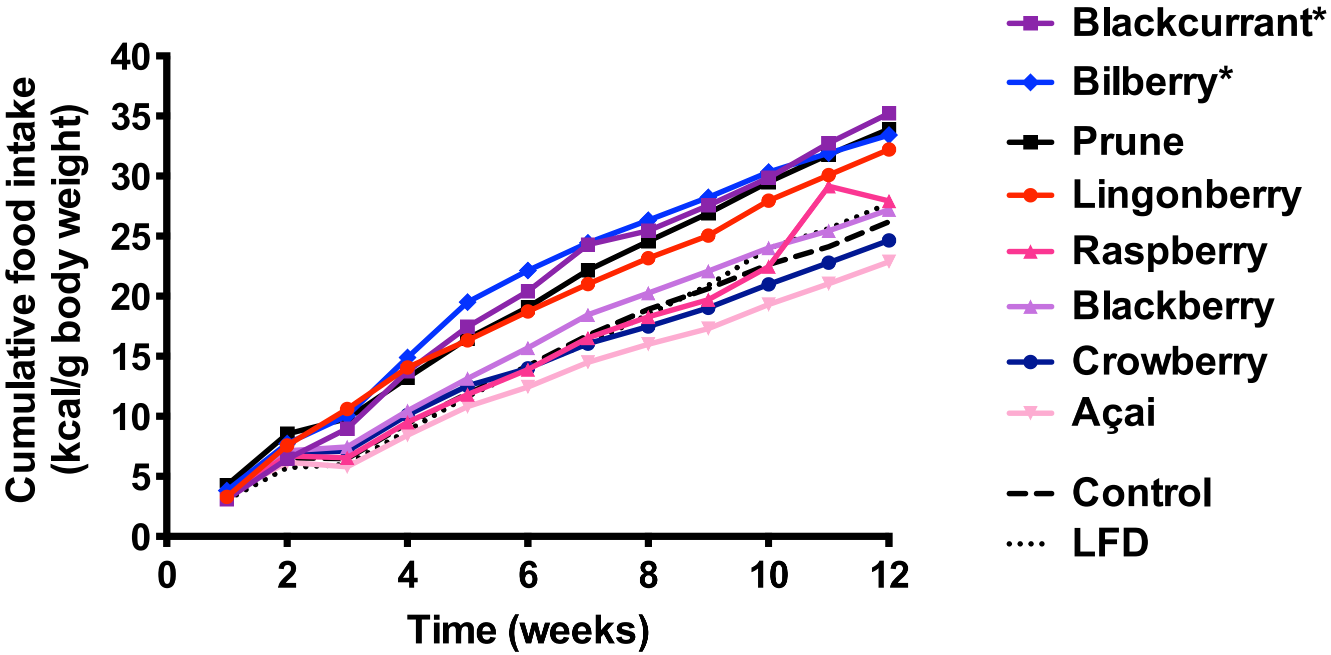

Figure S1. Cumulative food intake during 13 weeks of high-fat diet and berry supplementation. Weekly assessment of food intake in each cage (2 cages per group, 6 mice per cage) show increased consumption in the groups receiving blackcurrant and bilberry. Statistical comparisons are made to control using two-way ANOVA with Dunnet's post test, *$p<0.05$.
